# 3dCAP-Wheat: An Open-Source Comprehensive Computational Framework Precisely Quantifies Wheat Foliar, Nonfoliar, and Canopy Photosynthesis

**DOI:** 10.34133/2022/9758148

**Published:** 2022-07-21

**Authors:** Tian-Gen Chang, Zai Shi, Honglong Zhao, Qingfeng Song, Zhonghu He, Jeroen Van Rie, Bart Den Boer, Alexander Galle, Xin-Guang Zhu

**Affiliations:** ^1^National Key Laboratory for Plant Molecular Genetics, Center for Excellence in Molecular Plant Sciences, Chinese Academy of Sciences, Shanghai 200032, China; ^2^Insitute of Crop Sciences, Chinese Academy of Agricultural Sciences, Beijing 100081, China; ^3^International Maize and Wheat Improvement Center (CIMMYT) China Office, Chinese Academy of Agricultural Sciences, Beijing 100081, China; ^4^BASF Belgium Coordination Center-Innovation Center Gent, Technologiepark-Zwijnaarde 101, 9052 Gent, Belgium

## Abstract

Canopy photosynthesis is the sum of photosynthesis of all above-ground photosynthetic tissues. Quantitative roles of nonfoliar tissues in canopy photosynthesis remain elusive due to methodology limitations. Here, we develop the first *complete* canopy photosynthesis model incorporating all above-ground photosynthetic tissues and validate this model on wheat with state-of-the-art gas exchange measurement facilities. The new model precisely predicts wheat canopy gas exchange rates at different growth stages, weather conditions, and canopy architectural perturbations. Using the model, we systematically study (1) the contribution of both foliar and nonfoliar tissues to wheat canopy photosynthesis and (2) the responses of wheat canopy photosynthesis to plant physiological and architectural changes. We found that (1) at tillering, heading, and milking stages, nonfoliar tissues can contribute ~4, ~32, and ~50% of daily gross canopy photosynthesis (*A*_cgross_; ~2, ~15, and ~-13% of daily net canopy photosynthesis, *A*_cnet_) and absorb ~6, ~42, and ~60% of total light, respectively; (2) under favorable condition, increasing spike photosynthetic activity, rather than enlarging spike size or awn size, can enhance canopy photosynthesis; (3) covariation in tissue respiratory rate and photosynthetic rate may be a major factor responsible for less than expected increase in daily *A*_cnet_; and (4) in general, erect leaves, lower spike position, shorter plant height, and proper plant densities can benefit daily *A*_cnet_. Overall, the model, together with the facilities for quantifying plant architecture and tissue gas exchange, provides an integrated platform to study canopy photosynthesis and support rational design of photosynthetically efficient wheat crops.

## 1. Introduction

Plant canopy is defined as the sum of all the above-ground plant tissues, including both leaves and nonfoliar tissues (e.g., spikes and stems). The photosynthetic CO_2_ uptake of all these above-ground tissues together determines canopy photosynthesis. Canopy photosynthesis provides over 90% of the biomass for plant growth [1]. Improving canopy photosynthesis has been regarded as a major target to increase crop yield potential [2–4].

Though canopy photosynthesis comprises photosynthetic CO_2_ uptake of all photosynthetic tissues, most study focuses on leaf photosynthesis so far [5–9]. In contrast, photosynthesis of nonfoliar tissues, which are usually irregular in shape and heterogeneous in tissue composition, are much less studied, largely due to a lack of effective methodologies [10–12]. Recently, some new methods to study nonfoliar photosynthesis have been developed [13], such as isotope tracing [14], chlorophyll fluorescence measurement [15], or gas exchange measurements [16, 17]. However, these methods are in general semiquantitative as they cannot be used to provide a quantitative evaluation of the contribution of these different tissues to canopy photosynthesis.

Canopy architecture is another major factor influencing canopy photosynthesis [18–20]. Canopy architecture comprises many factors, such as plant height, plant density, spike length, spike height and position in the canopy, awn length, leaf size, leaf density, leaf thickness, leaf angle, and leaf number [21–24]. Some of these architectural parameters have already been manipulated in traditional crop breeding. For example, erect leaves have been selected by crop breeders as they may enhance light penetration into the bottom layer of a canopy and enable denser planting [25, 26]. Genes controlling leaf erectness in rice and maize have been cloned, and some alleles have been shown to increase biomass and grain production [27, 28].

In wheat, there is a positive correlation between awn length and contribution to grain filling; however, it is still not clear whether longer or shorter awns are preferred for higher canopy photosynthesis [11, 29, 30]. In brief, different architectural parameters may all contribute to canopy photosynthesis; however, their individual contributions and their optimal combinations for higher canopy photosynthesis still need to be systematically studied.

Systems modeling can be used to quantitatively assess the efficiency of photosynthesis and growth, to systematically identify the rate-limiting steps/targets, and to rationally design for higher efficiency [9, 31–34]. Various models of canopy photosynthesis with different levels of mechanistic details have been developed over the years since the first model by de Wit [35], such as the big-leaf model, the sunlit-shaded model, and the multilayer model [36].

These earlier models have a virtue of simplicity and can be relatively easily parameterized; however, they can hardly be used to support detailed analysis such as contribution of different tissues to canopy photosynthesis and design of optimal canopy architecture. The 3D canopy photosynthesis models can realistically simulate 3D canopy structure and heterogeneous microenvironments within a canopy [20, 37, 38]. However, photosynthesis of nonfoliar tissues is usually not explicitly represented in current models [39], which limits their application in studying canopy photosynthesis for such crops at the grain filling stage, which is crucial for final yield formation.

Here we introduced a three-dimensional canopy photosynthesis model of wheat (3dCAP-wheat), which incorporates all the above-ground plant photosynthetic tissues (i.e., leaves, stems, and spikes), and a set of measurement instruments and protocols for model validation. We use this fully parameterized model to predict canopy photosynthesis at any time of a day under different weather, to characterize the diurnal photosynthetic properties of different plant tissues and their contribution to canopy photosynthesis, and to systematically assess the impacts of major internal and external factors on canopy photosynthesis. Overall, this work provides an integrated platform to support canopy photosynthesis research, and rational crop design.

## 2. Materials and Methods

### 2.1. Field Experiments

To parameterize and validate the 3dCAP-wheat model, we thoroughly measured the 3D wheat plant architecture and photosynthetic physiology during the whole growth season. Field experiments were conducted at the Songjiang experimental station of Institute of Plant Physiology and Ecology, CAS, Shanghai, China (latitude 30°56′44″ N, 121°8′1″ E). Two elite Chinese wheat cultivars, Ning Mai 22 (N22) and Yang Mai 20 (Y20), were used for experiments. Seeds were sown on 1 November 2019 with a planting space between rows of 0.20 m and a sowing density of 240 seeds m^−2^. In total 18 plots of plants were grown, with a plot size of 1.5 m × 3.0 m.

Sampling were taken at three different developmental stages, i.e., the tillering stage, the heading stage, and the milking stage. Detailed plant morphological and physiological features were measured at each of these growth stages. Nitrogen, phosphate, and potassium fertilizers were applied at a rate of 100, 50, 50 kg ha^−1^ before sowing. Weeds, pests, and diseases were controlled periodically with herbicides, insecticides, and fungicides. The weather data, including photosynthetically active radiation (PAR), relative humidity, and air temperature, were recorded with a WatchDog 2900ET weather station (Spectrum Technologies, Inc. Aurora, IL, USA) every 10 minutes for the whole growing season.

### 2.2. Measurement of Photosynthetic Light Response Property of Leaf, Spike, and Stem

To simulate gas exchange rate of a plant tissue under certain incident light intensity, the photosynthetic light response property needs to be quantified. Leaf photosynthetic light response curves (*A-*Q curves) were measured using a LI-6400XT infrared analyzer (LI-COR Inc., Lincoln, Nebraska, USA) for all growth stages. Briefly, for each *A*-Q curve measurement, we first set the influx CO_2_ concentration as 400 *μ*mol mol^−1^, the air flow rate as 500 *μ*mol s^−1^, and the initial light levels as 2000 *μ*mol m^−2^ s^−1^; leaves were acclimated in the leaf chamber for 15 min; then light levels were changed to 2000, 1500, 1000, 700, 500, 300, 200, 100, 50, 25, and 0 *μ*mol m^−2^ s^−1^ with duration for each light level as 2 min.

*A-*Q curves of the intact spike and the stem (30 cm-long) were measured using a custom-built cuvette (with tunable artificial red-blue LED light source; P-Chamber) connected to a LI-6400XT infrared analyzer (Supplementary Fig. 1a) [16]. Briefly, for each *A*-Q curve measurement, we first set the influx CO_2_ concentration as 400 *μ*mol mol^−1^, the air flow rate as 680 *μ*mol s^−1^, and the initial total light level as 1500 *μ*mol m^−2^ s^−1^ (750 *μ*mol m^−2^ s^−1^ for both sides); the spike/stem was acclimated in the chamber for 15 min; then total light levels were changed to 2000, 1500, 1000, 700, 500, 300, 200, 100, 50, and 0 *μ*mol m^−2^ s^−1^ with duration for each light level as 5 min.

### 2.3. Measurement of Tissue Nitrogen Content

To build a relationship between tissue nitrogen content and photosynthetic light response parameters, we measured the nitrogen contents of matched tissues that were used to measure *A*-Q curves. After measuring an *A*-Q curve, plant tissue was sampled, dried and ground into powder for nitrogen content measurement. Nitrogen content (LNC) was measured with an elemental analyzer (vario ISOTOPE cube, Elementar, Hanau, Germany).

### 2.4. Temperature Responses of Tissue Photosynthetic Parameters

To predict photosynthetic rate of a plant tissue under different temperatures, which were further used to predict canopy photosynthetic rates under different temperatures, we measured the temperature responses of the net photosynthetic rate at a photosynthetic photon flux density of 1500 *μ*mol m^−2^ s^−1^ and the dark respiratory rate in a temperature-controlled growth room. We measured these temperature responses for leaf, stem, and spike separately. The room temperature was set at 7°C, 15°C, 22°C, and 28°C, respectively. The plants were adapted in each temperature for 30 min, and then the photosynthetic rate or respiratory rate was measured with the protocol as described above. During the measurement, we recorded the temperature in the cuvette enclosing each tissue.

### 2.5. Modeling Photosynthetic Rates of Individual Tissues

After having all the above measurements, the photosynthetic rate of every small piece of a plant tissue could be calculated with a nonrectangular hyperbola model:

(1) $$\begin{array}{c} \begin{array}{c} A\left(T\right)=\frac{\Phi_{\text{CO}2}\cdot I+A_{\max}\left(T\right)-\sqrt{{\left[\Phi_{\text{CO}2}\cdot I+A_{\max}\left(T\right)\right]}^{2}-4\cdot \theta \cdot \Phi_{\text{CO}2}\cdot I\cdot A_{\max}\left(T\right)}}{2\cdot \theta }-R_{d}\left(T\right),\\ A_{\max}\left(T\right)=A_{\max\_\text{net}}\left(T\right)+R_{d}\left(T\right),\\ A_{\max\_\text{net}}\left(T\right)=A_{\max\_\text{net}}\left(25\right)\cdot \left[a_{1}\cdot T^{2}+b_{1}\cdot T+c_{1}\right],\\ R_{d}\left(T\right)=R_{d}\left(25\right)\cdot \left[a_{2}\cdot T^{2}+b_{2}\cdot T+c_{2}\right], \end{array} \end{array}$$

where *A*(*T*) is the photosynthetic rate at temperature *T*; *A*_max_(*T*) and *R*_*d*_(*T*) are the gross light-saturated photosynthetic rate and the dark respiration rate at temperature *T*, respectively; *A*_max_net_(*T*) is the net light-saturated photosynthetic rate at temperature *T*; *I* is the absorbed photosynthetic photon flux density; *Φ*_CO2_ is the maximal apparent quantum efficiency of CO_2_ fixation; *θ* is the convexity of the nonrectangular hyperbola; *a*_1_, *b*_1_, *c*_1_, *a*_2_, *b*_2_, and *c*_2_ are empirical coefficients.

### 2.6. Modeling Wheat Canopy Architecture and Light Distribution

**Figure 1:**
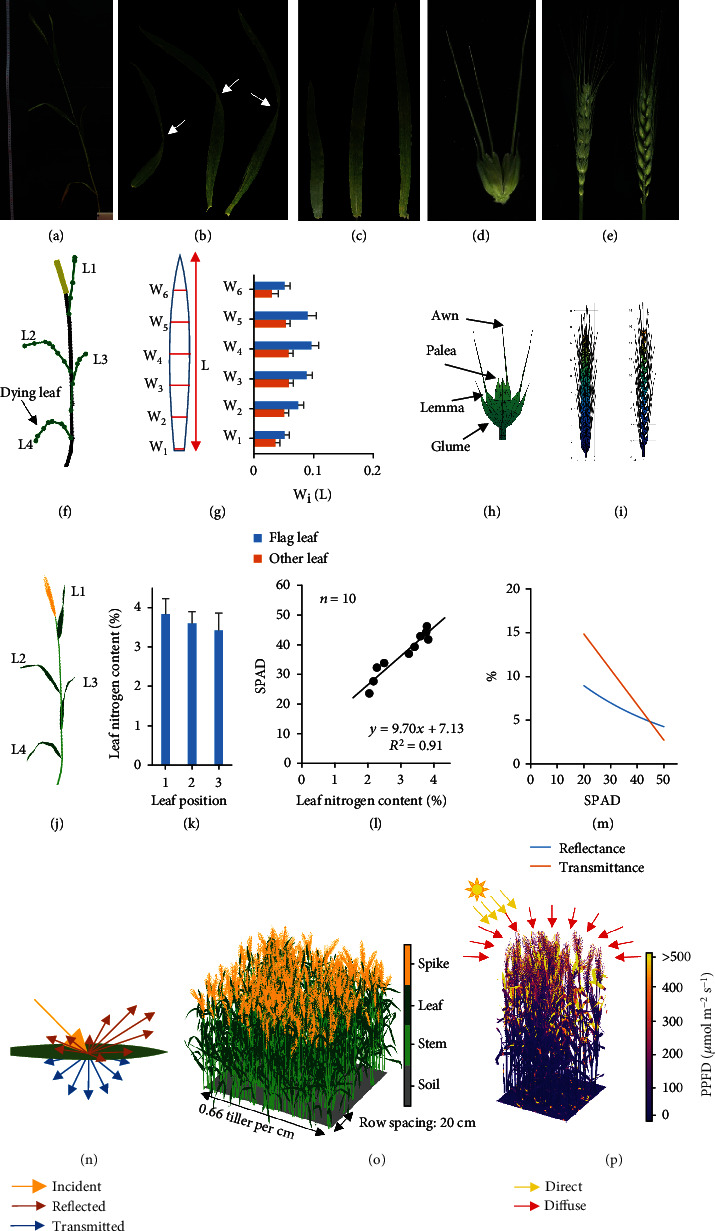
Modeling wheat canopy architecture and light distribution. (a–e) Photographs of a tiller, the natural status of the leaves on the tiller, the flat shapes of the leaves (by covering a glass), the spikelets, and the front-view and side-view of the spike. (f–i) Reconstructed tiller skeleton, flat leaf shape, 3D spikelet, and 3D spike. (j) 3D reconstruction of a tiller. (k) Nitrogen contents of leaves on different positions of a tiller. (l) Relationship between leaf nitrogen content and leaf chlorophyll content (SPAD values). (m) Modeling leaf reflectance and transmittance based on the SPAD value. (n–p) An illustration of light reflectance and transmittance profiles, the 3D reconstructed canopy, and the light distribution within the canopy. Data measured from cultivar N22 were used.

To obtain a complete set of parameters for plant 3D reconstruction, we developed a pipeline to extract plant architectural parameters based on imaging and image processing:

1. Estimate maximal tiller angle *α*0 for plants in the field
2. Randomly choose a position in a plot, and harvest 10 neighboring tillers from the base of the stem at that position
3. Photograph the sampled tillers one by one (Figure 1(a)). Tips: put a scale aside the tiller and keep a fixed position and a fixed focal length of the camera during photographing (the same below)
4. Detach the leaves on the tillers, arrange them in order, and photograph them in their natural status (to obtain the twisting profile; Figure 1(b)), and then cover the leaves with a glass plate, and photograph the flattened leaves to characterize their 2D shape (Figure 1(c))
5. Detach the spikes, hold them horizontally, and photograph them from the front view and the side view, respectively (Figure 1(e))
6. Detach the spikelets, arrange them by their orders on the ear, and scan them with a scanner from the front view with a resolution of 1200 dpi (Figure 1(d))

The tiller skeleton can be reconstructed from the information from the step (3) (Figure 1(f)), the 2D (Figure 1(g)) and 3D leaf can be reconstructed from information from the step (4), and the 3D spikelet and spike can be reconstructed from information from steps (5)-(6) (Figures 1(h) and 1(i)). The reconstructed 3D individual tissues comprise small triangular patches (e.g. Figures 1(h) and 1(i)). The 3D tiller comprising numerous triangular patches was finally reconstructed by mapping the 3D structure of a spike and leaves to the skeleton of the tiller (Figure 1(j)). A series of scripts were developed to extract architectural parameters from the above acquired images for plant 3D reconstruction (https://github.com/rootchang/3dCAP-wheat).

To simulate the optical property of each tissue, we firstly measured nitrogen contents of leaves at different positions on a tiller (Figure 1(k)). The leaf nitrogen content was then used to calculate the chlorophyll content (SPAD values) based on an empirical relationship between the leaf nitrogen content and leaf SPAD (%, dry weight; Figure 1(l); *R*^2^ = 0.91).

(2) $$\begin{array}{c} \text{SPAD}=9.7\cdot \text{LNC}+7.13. \end{array}$$

The transmittance and reflectance of a “leaf facet” were modeled based on its chlorophyll content by fitting the PROSPECT-5 model [40]:

(3) $$\begin{array}{c} K_{r}=0.3605\cdot {\left[\text{chl}\right]}^{\left(-0.502\right)},\\ K_{t}=‐0.082\cdot \log\left(\left[\text{chl}\right]\right)+0.3761, \end{array}$$

in which *K*_*r*_ and *K*_*t*_ are reflected and transmitted proportion of total incident light on the leaf and [chl] is leaf chlorophyll concentration (*μ*g cm^−2^). The wheat leaf chlorophyll concentration is exponentially correlated with its SPAD value [41]:

(4) $$\begin{array}{c} \left[\text{chl}\right]=0.0599\cdot e^{0.0493\cdot \text{SPAD}}\cdot 100. \end{array}$$

As a result, light transmittance and reflectance of plant tissues were determined based on the SPAD values (Figure 1(m)). Reflectance of spike and stem were modeled in the same way. Transmittance of spike and stem were set to 0. Spatial distribution of light reflection is modeled with Cook-Torrance bidirectional reflectance distribution function (BRDF), and spatial distribution of light transmittance is modeled with Lambert bidirectional transmittance distribution function (BTDF) with a Monte Carlo method as described before [20] (Figure 1(n)).

Briefly, In the ray tracing, ambient incident light includes direct light and diffuse light. The direct light was represented with parallel rays and diffuse light with random rays. Once a ray hit a leaf, a reflect ray and a transmit ray were emitted, and we need to calculate their direction and light intensity. The direction of the reflect ray was randomized with Monte Carlo method as the distribution of Cook-Torrance BRDF, while the direction of the transmit ray with Lambert distribution. The light intensities of the reflect ray and transmit ray were calculated based on the leaf reflectance and transmittance predicted with SPAD values. Then, the reflect ray and transmit ray continued transferring in the canopy and hit possible leaves until the light intensity of the rays was lower than a threshold (1 *μ*mol m^−2^ s^−1^).

Finally, given the tiller density in a row and the distance between two rows, a virtual wheat canopy was reconstructed (Figure 1(o)). Furthermore, using a previously developed forward ray-tracing algorithm [20], the light distribution within the synthetic canopy was simulated (Figure 1(p)).

### 2.7. Construction of the 3dCAP-Wheat Model for Different Growth Stages

After modeling photosynthetic rate of individual tissues and reconstruction of the 3D wheat canopy architecture, we constructed the 3dCAP-wheat model. The detailed protocol can be found in the User Manual on the *GitHub*. Briefly, we

1. randomly sample 10 neighboring tillers from 5 independent plots and take photos of them (and the plant tissues) for plant architecture extraction
2. reconstruct 3D architecture of these 50 tillers to form a “virtual-tiller library”
3. determine distance between rows and plant density in a row
4. randomly pick a tiller from the library and randomly arrange it in a row with a random tiller angle between zero and the observed maximal tiller angle, and repeat the process until reaching the measured plant density
5. measure the photosynthetic light/temperature/nitrogen response curves and calculate the photosynthetic light/temperature/nitrogen response parameters of different plant tissues (i.e., leaves, stems, and spikes)
6. simulate light distribution inside the virtual canopy with the input environmental parameters and parameters of leaf transmittance and reflectance using a forward ray-tracing algorithm *fastTracer* [20], and calculate canopy photosynthetic CO_2_ uptake rate as a sum of photosynthetic rate of all individual tissues, where the photosynthetic rates of individual tissues are calculated using the nonrectangular hyperbola equations

### 2.8. Development and Validation of a Ray-Tracing-Based Nonfoliar Photosynthesis Model

**Figure 2:**
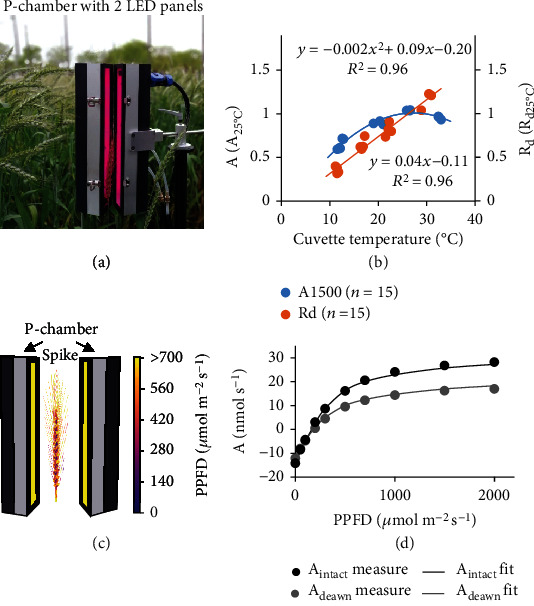
Characterization of wheat spike photosynthesis. (a) A custom-built P-Chamber to measure gas exchange rates of nonfoliar tissues. (b) Temperature response pattern of relative photosynthetic and respiratory rates of a spike. (c) Simulated light distribution within a spike enclosed in a P-Chamber. (d) The measured and simulated photosynthetic light response curves of the intact and deawned spikes.

We developed a ray-tracing-based method to model photosynthesis of nonfoliar tissues with irregular 3D shapes, such as a spike or a stem. Using wheat spike as an example, we firstly measured the gas exchange rates of spikes using a custom-built chamber, named as P-Chamber, which has two independent programmable LED panels (both with red/blue lamp beads; light profile of red: blue = 90%: 10% was used during measurement) illuminating the enclosed plant tissues from two opposite directions (Figure 2(a)). The P-Chamber is capable of measuring gas exchange rates of irregular nonfoliar tissues (e.g., spikes and stems) by connecting to an open-path infrared gas analyzer [16].

We further measured the spike photosynthetic rate (under a light level of 1500 *μ*mol m^−2^ s^−1^, *A*_1500_) and respiratory rate (*R*_*d*_) under different temperatures in a temperature-controlled growth room (Figure 2(b)). We obtained highly significant correlation between temperature and either *A*_1500_ (*R*^2^ = 0.96) and *R*_*d*_ (*R*^2^ = 0.96) (Figure 2(b)).

Then, we calculated light interception of each small patch of the spike by reconstructing the 3D spike and performing ray tracing to simulate the light distribution in the P-Chamber (Figure 2(c)). For each small patch of the spike, we used the classical nonrectangular hyperbola equation to calculate its photosynthetic CO_2_ uptake rate under its incident light level (*I*) and the air temperature (*T*) (see section “Modeling photosynthetic rate of individual tissues” in the above).

There are four photosynthetic parameters of the nonrectangular hyperbola model, i.e., the net saturated photosynthetic rate (*A*_max_net_), the dark respiration rate (*R*_*d*_), the maximal apparent quantum efficiency of CO_2_ fixation (*Φ*_CO2_), and the convexity of the nonrectangular hyperbola (*θ*). The awn and the nonawn tissues of spikes had different sets of photosynthetic parameters. With the measured light response curves of a spike (*A*_intact_) before and after removing its awns (*A*_deawn_) (Figure 2(d)), the eight photosynthetic parameters (four for the awn tissues and the other four for the nonawn tissues) were solved iterately. Experimentally, we thoroughly parameterized and validated the model as follows (Supplementary Fig. 2):

Day 1: pick up a spike in the field and measure its *A*-Q curve using the P-Chamber. At 18 : 00, enclose the spike with a transparent cuvette, and connect the transparent cuvette to the Li-6400 to monitor the spike gas exchange rate every 20 seconds for the whole night

Day 2: keep monitoring the gas exchange rate of the spike by the transparent cuvette. Cover the transparent cuvette by a silver cloth around 9 : 00, 12 : 00, 15 : 00, and 17 : 30 for 20 min to measure the diurnal spike respiration. At 18 : 00, remove the transparent cuvette, and remove the awns on the spike carefully by a surgical scissor

Day 3: measure the *A*-Q curve of the deawned spike using a P-Chamber. At 18 : 00, enclose the spike with the transparent cuvette, and connect the transparent cuvette to the Li-6400 to monitor gas exchange rate of the deawned spike every 20 seconds for the whole night

Day 4: keep monitoring the gas exchange rate by the transparent cuvette. Cover the transparent cuvette by a silver cloth around 9 : 00, 12 : 00, 15 : 00, and 17 : 30 for 20 min to measure the diurnal respiration of the deawned spike. At 18 : 00, remove the transparent cuvette. Then, harvest the deawned spike, and measure the spike architectural parameters.

Both the P-Chamber and the transparent cuvette were equipped with high-precision temperature and humidity sensors, which recorded air temperature and humidity in the cuvette during measurement of gas exchange rate. For data analysis, the wheat spike *A*-Q curves measured on the 1^st^ day and the 3^rd^ day (with and without awn, respectively) were used for parameterizing the photosynthetic light response parameters. Daily dynamic wheat spike gas exchange rates measured by the transparent cuvettes on the 2^nd^ day and the 4^th^ day (with and without awn, respectively) were used to validate the model.

### 2.9. Validation of the 3dCAP-Wheat Model

To validate the gas exchange rate predicted by the 3dCAP-wheat model, the canopy gas exchange rate was measured directly. The canopy photosynthetic and respiratory rates of the 1 m∗1 m canopies were measured automatically every 10 min for the 2 wheat cultivars using the canopy photosynthesis and transpiration system (CAPTS). CAPTS consists of a cubic transparent chamber, which can be open and closed automatically with programming, and is equipped with CO_2_, humidity, air pressure, and temperature sensors in it (Supplementary Fig. 1b). CAPTS measures the canopy gas exchange rate by monitoring the CO_2_ concentration change rate in the chamber during the closure of the chamber [42].

To further validate the predictions of the 3dCAP-wheat model, we performed two additional treatments. The first one was the spike removal experiment, which was used to validate whether spike removal during the milking stage could indeed increase canopy photosynthetic rate. During the milking stage, six subplots each with a size of 1.0 m∗1.0 m were randomly chosen. In three of the subplots, all spikes were removed manually. Daily dynamic canopy gas exchange of the six subplots were recorded with the CAPTS before and after spike removal.

The second treatment was scattering-film covering treatment, which was used to validate whether more scattering light could increase canopy photosynthetic rate of a closed wheat canopy. During the heading stage, nine subplots each with a size of 1 m∗1 m were randomly chosen. In three of the subplots, a PVC film with 90% transmittance was used to cover on the top of the CAPTS; in another three of the subplots, an optical scattering film with a transmittance of 90% and a haze of 50%, i.e., changing 50% of the direct incident light into scattering light, was covered on the top of the CAPTS. Daily dynamic canopy gas exchange rates of the nine subplots were recorded with CAPTS before and after covering the films.

## 3. Results

### 3.1. A Ray-Tracing-Based Model Quantifies Gas Exchange of Foliar and Nonfoliar Tissues in a Canopy

The 3dCAP-wheat comprises two parts, i.e., the structural part and the functional part. The structural part of the 3dCAP-wheat simulates detailed 3D wheat canopy architecture and light distribution. Detailed procedures of 3D canopy reconstruction can be found in the Materials and Methods section. In the functional part of the 3dCAP-wheat, we modeled the photosynthetic CO_2_ uptake rate of each small patch of foliar and nonfoliar tissues. The major challenges here are the measurement and modeling of the 3D architecture, light distribution, and photosynthesis of the nonfoliar tissues.

To overcome these challenges, we firstly measured the gas exchange rates of the nonfoliar tissues using a custom-built P-Chamber [16]. Then, for each small patch of the tissue, we calculated its light interception by reconstructing tissue 3D architecture and performing ray tracing to simulate the light distribution in the P-Chamber. We further used the classical nonrectangular hyperbola model to calculate its photosynthetic CO_2_ uptake rate under its incident light level (*I*) and the cuvette temperature (*T*). With the measured *A*-Q curves, the photosynthetic parameters (i.e., *A*_max_net_, *R*_*d*_, *Φ*_CO2_, and *θ*) were solved iterately (see details in the Materials and methods).

**Figure 3:**
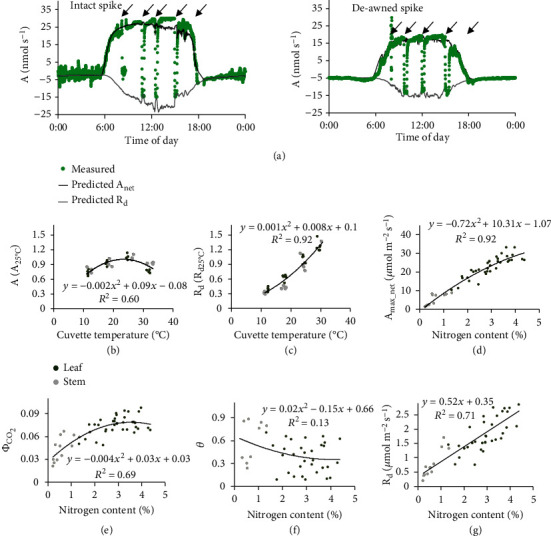
A ray-tracing-based model characterizing gas exchange of foliar and nonfoliar tissues in a canopy. (a) The measured and simulated daily dynamic gas exchange rates of the intact and deawned spikes. (b and c), temperature response patterns of leaf/stem photosynthetic and respiratory rates (normalized to 1 at 25°C). Photosynthetic rates were measured under a light level of 1500 *μ*mol m^−2^ s^−1^. (d–g), relationship between tissue nitrogen content and the photosynthetic parameters. The four photosynthetic parameters are the net saturated photosynthetic rate (*A*_max_net_), the maximal apparent quantum efficiency of CO_2_ fixation (*Φ*_CO2_), the convexity of the nonrectangular hyperbola (*θ*), and the dark respiration rate (*R*_*d*_). Data measured from cultivar N22 were used. Arrows in panel (a) illustrate time when spike respiratory rates were measured.

To confirm the validity of this new method to quantify photosynthesis of nonfoliar tissues, especially those with irregular shapes, we further recorded the 24-hour gas exchange rates of a spike with a transparent cuvette together with the real-time temperature and moisture in the cuvette before and after removal of its awns (Supplementary Fig. 3). At the same time, we predicted the 24-hour gas exchange rates of spikes with and without awns by reconstructing the 3D spike, simulating the light distribution, correcting temperature effect, and calculating the gas exchange rates with correction of the temperature effect at different times of a day. Remarkably, the new method realistically predicted the 24-hour dynamic gas exchange rates (net photosynthetic rate *A*_net_ and dark respiratory rate *R*_*d*_) for both the intact (*R*^2^ = 0.83 for *A*_net_, and *R*^2^ = 0.61 for *R*_*d*_) and the deawned (*R*^2^ = 0.96 for *A*_net_, and *R*^2^ = 0.95 for *R*_*d*_) spikes (Figure 3(a)).

After demonstrating the validity of the new method, we used the same strategy to model the gas exchange of a stem. We first characterized the temperature response functions for photosynthetic and respiratory rates of a leaf and a stem (Figures 3(b) and 3(c)). We further derived relationships between the photosynthetic light response parameters (i.e., *A*_max_net_, *Φ*_CO2_, *θ*, and *R*_*d*_) and tissue nitrogen content (Figure 3(d)–3(g); *R*^2^ = 0.92, 0.69, 0.13, and 0.71, respectively), which were used to calculate leaf and stem photosynthetic rates under different light conditions based on the measured tissue nitrogen contents at different growth stages.

### 3.2. The 3dCAP-Wheat Precisely Predicts Canopy Gas Exchange under Multiple Scenarios

**Figure 4:**
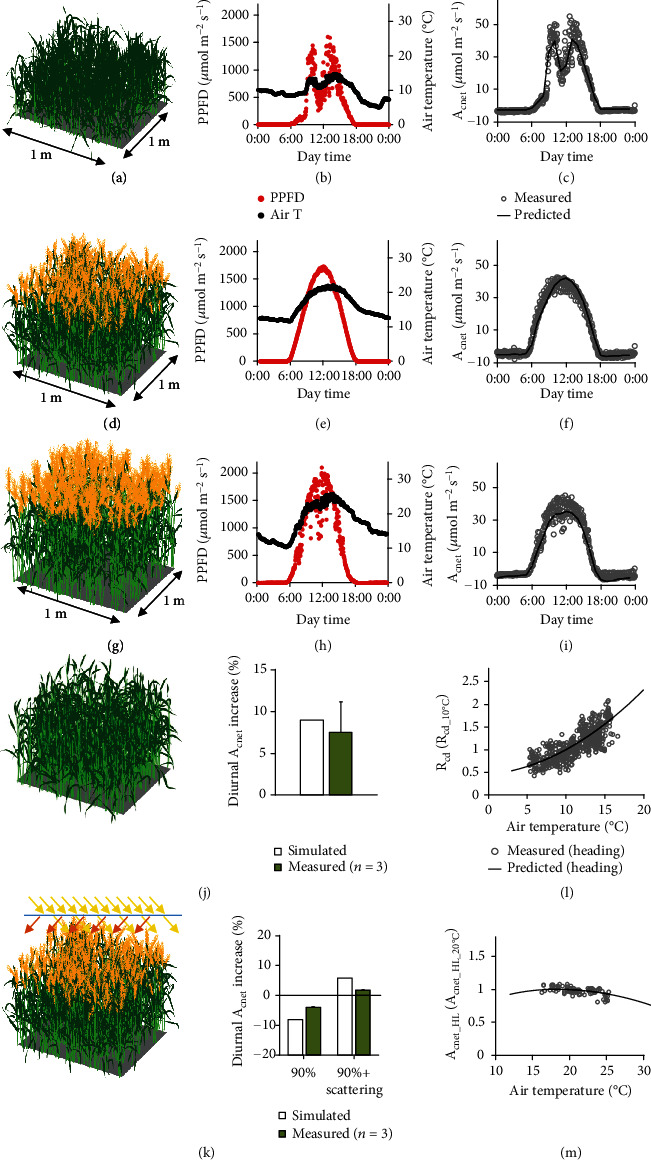
A comprehensive validation of the 3dCAP-wheat canopy photosynthesis model by precise predicting canopy gas exchange at different growth stages, under different weather, and under different perturbations. (a–c) Canopy architecture, weather, and canopy photosynthesis at the tillering stage. (d–f) Canopy architecture, weather, and canopy photosynthesis at the heading stage. (g–i) Canopy architecture, weather, and canopy photosynthesis at the grain milk stage. (j) Increase of the diurnal net canopy photosynthesis by removing the spikes. (k) Changes of the diurnal net canopy photosynthesis by top-covering a PVC film with 90% transmittance and top-covering a scattering film with 90% transmittance and 50% scattering. (l and m) Temperature responses of canopy night respiratory rate (*R*_cd_) and canopy photosynthetic rate under high light (*A*_cnet_HL_; PPFD >1500 *μ*mol m^−2^ s^−1^) at the heading stage. Note that canopy night respiratory rate was normalized to 1 at 10°C (*R*_cd_10oC_), while canopy photosynthetic rate under high light was normalized to 1 at 20°C (*A*_cnet_HL_20oC_). Data measured and simulated for cultivar N22 were used.

To further test the performance of the 3dCAP-wheat, we reconstructed wheat canopies for three different growth stages, i.e., the tillering stage (Figure 4(a)), the heading stage (Figure 4(d)), and the grain milk stage (Figure 4(g)). The size of the canopy is 1 m width∗1 m length. To avoid the border effect, we performed ray tracing and canopy photosynthetic rate calculation in the central 0.4 m∗0.4 m region.

Experimentally, we recorded the 24-hour dynamic canopy gas exchange rates with a custom-built canopy photosynthesis and transpiration measurement system. Finally, the 3dCAP-wheat was tested on an overcast day at the tillering stage (Figure 4(b)), a sunny day at the heading stage (Figure 4(e)), and a cloudy day at the grain milk stage (Figure 4(h)). Remarkably, the 3dCAP-wheat successfully predicted the 24-hour dynamic canopy gas exchange rates for all these three stages (Figures 4(c), 4(f), and 4(i)).

We further tested the model performance under two perturbations. First, we simulated the influence of removing spikes on canopy photosynthesis. Surprisingly, we predicted that the canopy net photosynthesis can be increased by 9% if all spikes are removed at the grain milk stage (Figure 4(j)). Consistently, field experimental results show that canopy photosynthesis was indeed increased by 7.5 ± 3.6% (mean ± sd; *n* = 3) when the spikes were removed (Figure 4(j)).

Second, we studied the impacts of direct/diffuse ratio of incident light on canopy photosynthesis. We calculated the canopy photosynthesis of the field-grown plants under three scenarios: (1) without cover, (2) covering with a normal PVC board (with 90% transparency), and (3) covering with a scattering film, which can transmit 90% of incident light and convert 50% of direct incident light into scattering light. Our results show that daily canopy photosynthetic CO_2_ uptake covered with scattering film was increased by 2 ± 3%, whereas that of plants covered with a normal PVC board was decreased by 4 ± 2%. These measured change trends of canopy photosynthesis were consistent with the model predictions (6% and -8%, respectively) (Figure 4(k)).

Lastly, we tested if the 3dCAP-wheat can predict canopy level temperature responses of photosynthetic rates and respiratory rates based on the tissue level temperature response functions (Figures 2(b) and 3(b) and 3(c)). We collected the canopy net photosynthetic rates when incident photosynthetic photon flux density (PPFD) exceeds 1500 *μ*mol m^−2^ s^−1^ (*A*_cnet_HL_) and canopy respiratory rates at night (*R*_cd_) under different air temperature at the heading stage. Remarkably, the model precisely reproduced the rapid increase of *R*_cd_ with the increase of temperature from 5°C to 15°C (Figure 4(l)).

Surprisingly, although *A*_1500_ keeps increasing rapidly until 25°C at a single tissue scale (Figures 2(b) and 3(b)), the change pattern of *A*_cnet_HL_ versus air temperature differed dramatically at canopy level. Specifically, the relative change of *A*_cnet_HL_ versus air temperature was unexpectedly small; more surprisingly, a decreasing trend in *A*_cnet_HL_ was predicted when air temperature exceeds 18°C, rather than 25°C (Figure 4(m)). Further analysis showed that a majority of plant tissues is under low light intensity, even at noon (Supplementary Fig. 4). Namely, under higher air temperature, photosynthetic rates of only a small proportion of (well-lighted) plant tissues in the canopy increase, while the respiratory rates of all plant tissues increase, which together compromise the overall canopy photosynthetic CO_2_ uptake rate.

### 3.3. Nonfoliar Tissues Contribute 4~50% Daily Gross Canopy Photosynthesis (-19~16% Daily Net Canopy Photosynthesis) and Take up 6~61% of Total Light Absorbed by a Canopy

Using the validated 3dCAP-wheat, we evaluated a number of factors influencing canopy photosynthesis, e.g., different photosynthetic tissues, spike traits, leaf nitrogen content, and plant architecture. Although qualitatively these factors have long been recognized as important players influencing wheat yield, their quantitative impacts on canopy photosynthesis have not been systematically evaluated.

**Figure 5:**
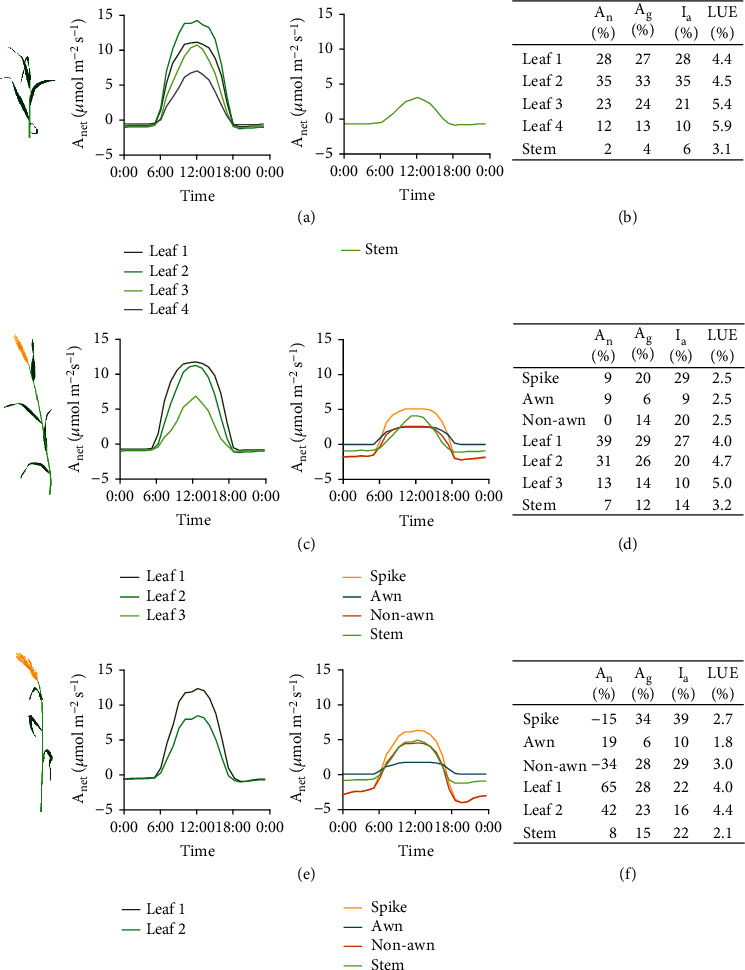
A landscape of photosynthetic characteristics of different foliar and nonfoliar tissues in the canopy. The 24-hour dynamic net gas exchange rate of different tissues in the canopy at the tillering (a), heading (c), and grain milk (e) stages on typical sunny days. The daily net photosynthesis, daily gross photosynthesis, daily light absorption, and daily light use efficiency (LUE) of different tissues at the tillering (b), heading (d), and grain milk (f) stages on typical sunny days. *A*_*n*_ (%): the ratio between tissue daily net photosynthesis and canopy daily net photosynthesis; *A*_*g*_ (%): the ratio between tissue daily gross photosynthesis and canopy daily gross photosynthesis; *I*_*a*_ (%): the ratio between tissue daily light absorption and canopy daily light absorption; LUE: the ratio between the daily gross photosynthesis and the daily light absorption. The cultivar N22 was used.

First, we estimated the contributions of different photosynthetic tissues to canopy photosynthetic CO_2_ uptake rate. We tackled this problem by reconstructing the canopy architecture and simulating canopy gas exchange of different tissues with the 3dCAP-wheat (Figures 5(a), 5(c), and 5(e); Supplementary Fig. 5a, c, e; see weather information in Supplementary Fig. 6).

We firstly calculated the contribution of each tissue to daily total canopy photosynthesis (Figures 5(b), 5(d), and 5(f); Supplementary Fig. 5b, d, f). As expected, the uppermost two leaves are the major contributor of canopy photosynthesis at all three growth stages, which contributed 51~60% and 50~60% of daily *A*_cgross_ for wheat cultivar N22 and Y20, respectively. Among nonfoliar tissues of the two cultivars, we found that the stem contributed 4%, 12~14%, and 15~20% of daily *A*_cgross_ at the tillering, heading, and grain milk stages, respectively, whereas the spike contributed 18~20% (with 6% from the awn and 12~14% from the nonawn tissues) and 30~34% (with 6% from the awn and 24~28% from the non-awn tissues) of daily *A*_cgross_ at the heading and grain milk stages, respectively.

Taking together, the nonfoliar tissues contributed 4%, 32%, and 49~50% of daily *A*_cgross_ at the tillering, heading, and mid-grain-filling stages, respectively. However, it is worth noting that in terms of daily *A*_cnet_, the nonfoliar tissues contributed much less to canopy photosynthesis, which were 2%, 15~16%, and -19~-7% at the tillering, heading, and grain milk stages, respectively (Figures 5(b), 5(d), and 5(f); Supplementary Fig. 5b, d, f).

We further calculated the daily light absorption by different tissues (Figures 5(b), 5(d), and 5(f); Supplementary Fig. 5b, d, f). Our results show that leaves absorbed 94%, 57~58%, and 39~40% of the total absorbed light by the canopy at the tillering, heading, and grain milk stages, respectively. Spikes, which are located at the top of the canopy, absorbed 26~29% (with 9% absorbed by the awn and 17~20% absorbed by the nonawn tissues) and 36~39% (with 10~11% absorbed by the awn and 25~29% absorbed by the non-awn tissues) at the heading and grain milk stages, respectively. On the contrary, stems absorbed a relatively small proportion of light, i.e., 6%, 14~15%, and 22~23% at the tillering, heading, and grain milk stages, respectively. Taken together, the nonfoliar tissues absorbed 6%, 41~43%, and 59~61% canopy light at the three stages, respectively.

In addition, we calculated the daily light use efficiency (LUE) of different tissues (Figures 5(b), 5(d), and 5(f); Supplementary Fig. 5b, d, f). Here the LUE for a tissue is defined as the ratio between the daily *A*_cgross_ (*μ*mol m^−2^ day^−1^) and total solar radiation absorption (*μ*mol m^−2^ day^−1^) by this tissue. Throughout the tillering, heading, and grain milk stages, leaves had the highest LUE of 3.9~6.3%, followed by the stem, which had a LUE of 2.1~3.6%. Spike had the lowest LUE of 2.5~2.7% (with the awn LUE of 1.8~2.5% and the nonawn LUE of 2.5~3.0%).

### 3.4. Canopy Photosynthesis Can Be Enhanced by Increasing Spike Photosynthetic Activity rather than by Elongating Awn Length or Spike Length under Favorable Condition

**Figure 6:**
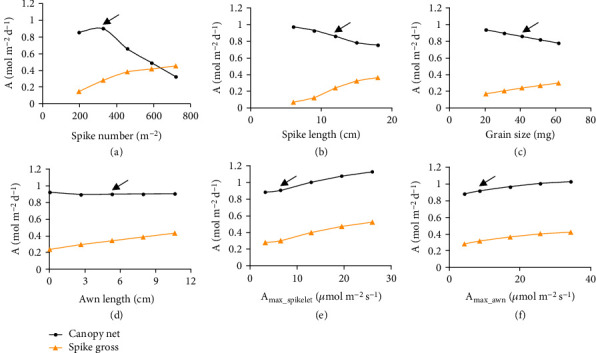
The change pattern of daily *A*_cnet_ and daily spike gross photosynthesis with change in different spike traits. The spike traits are spike number (a), spike length (b), spikelet size (c), awn length (d), maximal photosynthetic capacity of the spikelets (e), and maximal photosynthetic capacity of the awns (f). The arrows show the real values of traits in wheat cultivar N22.

Spike photosynthesis has been regarded as a major contributor of wheat yield. To study influence on canopy photosynthesis of different approaches that increase spike photosynthesis, we evaluated the impacts of modifying different spike properties on wheat spike and canopy photosynthesis. There are several potential approaches to increase spike photosynthesis: (1) higher spike number, (2) longer spike with more spikelets, (3) larger spikelets, (4) longer awn, and (5) higher spike photosynthetic activity. As a result, we found that all these factors can be used to increase spike daily gross photosynthesis (Figure 6). However, these factors had drastically different impact on canopy net photosynthesis.

First, a quadratic relationship was found between daily *A*_cnet_ and spike number (Figure 6(a)). Namely, daily *A*_cnet_ first increases then decreases with an increase in tiller number, due to the saturation of *A*_cgross_ and the continued increase in respiratory rate when the tiller number is higher than the optimum.

Second, daily *A*_cnet_ decreased with both longer spike and larger spikelets (Figures 6(b) and 6(c)), due to the lower LUE of spikes and their shading effect on leaves. As an extreme case, when all the spikes at the grain milk stage were removed, the diurnal *A*_cnet_ (6 : 00-18 : 00) can be increased by 9% (Figure 4(j)).

Third, although light interception and daily accumulated photosynthesis of almost all individual tissues in the canopy were affected by awn removal/elongation (Supplementary Fig. 7), only negligible influence was found on the daily *A*_cnet_ (Figure 6(d)).

Finally, unsurprisingly, daily *A*_cnet_ was higher when the photosynthetic activities of both spikelets and awns were higher (Figures 6(e) and 6(f)). Specifically, if the light saturated gross photosynthetic rates of the spikelet or awn can be increased to ~25 *μ*mol m^−2^ s^−1^, a level similar to that of the flag leaf, the daily *A*_cnet_ may be increased by ~25% or ~10%, respectively (Figures 6(e) and 6(f)).

### 3.5. Covariation in Tissue Respiratory Rate and Photosynthetic Rate May Be a Key Factor Compromising the Conversion from Leaf Level Photosynthetic Capacity to Daily *A*_cnet_ Gain

Increased application of nitrogen fertilizer has been regarded as one of key factors that improve crop growth and yield since the Green Revolution. To study the implications of tissue nitrogen content to canopy photosynthesis, we evaluated the impact of modifying leaf nitrogen content on canopy photosynthetic rate. Firstly, we found leaf nitrogen content highly influenced its gas exchange parameters (Figures 3(d)–3(g)). Given higher nitrogen content can increase both photosynthetic rate and respiratory rate (Figures 3(d) and 3(g)), we evaluated the impact of modifying leaf nitrogen content on canopy photosynthetic rate.

**Figure 7:**
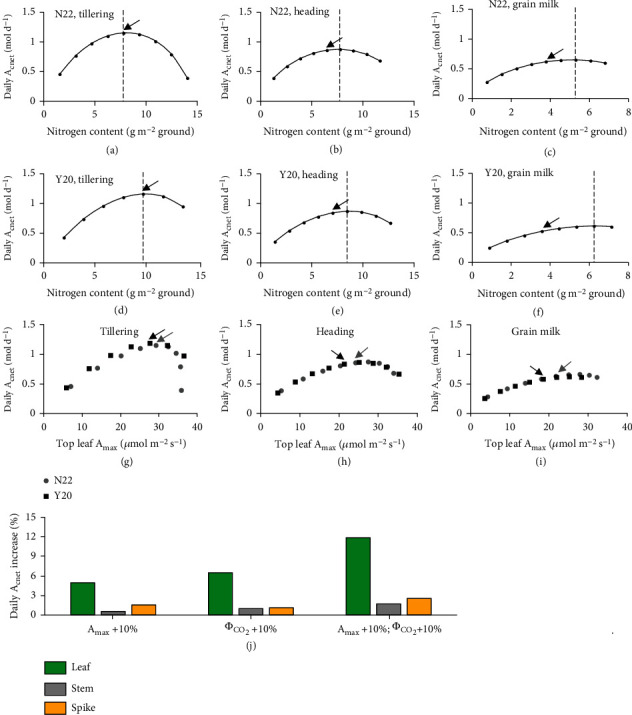
The conversion from tissue level photosynthetic capacity enhancement to canopy level daily net photosynthetic gain is dependent on covariation in tissue respiratory rate. (a–c) Change in daily net canopy photosynthesis with different leaf tissue nitrogen content at the tillering stage (a), the heading stage (b), and the grain milk stage (c) for wheat cultivar N22. (d–f) Change in daily net canopy photosynthesis with different leaf tissue nitrogen content at the tillering stage (d), the heading stage (e), and the grain milk stage (f) for wheat cultivar Y20. (g–h) The relationship between daily net canopy photosynthesis and maximal photosynthetic capacity of the uppermost leaves at the tillering stage (g), the heading stage (h), and the grain milk stage (i) for both cultivars. (j) The increase in daily net canopy photosynthesis by 10% increase of leaf/stem/spike maximal photosynthetic capacity (*A*_max_), apparent quantum efficiency (*Φ*_CO2_), or both at the heading stage. The arrows show the real values of traits in wheat cultivar N22 or Y20.

Our results show that, at a low nitrogen level, the increase of leaf nitrogen content increases daily *A*_cnet_ for both cultivars (Figures 7(a)–7(f)). However, when the leaf nitrogen content is higher than an optimal level, further increase in leaf nitrogen content decreases daily *A*_cnet_ (Figures 7(a)–7(f)). This is because the increase in respiration is higher than the increase in photosynthesis at the canopy level. The optimal leaf nitrogen contents differ between cultivars and between different developmental stages, which depend on canopy architecture (dashed lines in Figures 7(a)–7(f)). More intriguingly, from these synthetic data, we found nonlinear relationships between photosynthetic capacity of the uppermost leaves (*A*_max_leaf_) and daily *A*_cnet_ (Figures 7(g)–7(i)).

However, what if *A*_max_net_ and/or *Φ*_CO2_ could be improved without an associated increase in *R*_*d*_? When looking at the cultivar N22 at the heading stage, simulations show that at the canopy scale, a 10% increase in leaf *A*_max_net_ only converted to 4.9% increase in daily *A*_cnet_; a 10% increase in leaf *Φ*_CO2_ converted to 6.4% increase in daily *A*_cnet_, which is 31% (6.4/4.9-1) more than that of increase in *A*_max_net_ (Figure 7(j)). In contrast, for spike, a 10% increase in *A*_max_net_ converted to 1.56% increase in daily *A*_cnet_, which is 39% (1.56/1.12-1) more than that of increase in *Φ*_CO2_ (Figure 7(j)). This difference between leaf and spike is due to that most of the leaves in a canopy are under low light, whereas spikes are usually under high light. In addition, when the *A*_max_net_ and *Φ*_CO2_ of leaves, spikes, and stems can be increased simultaneously, the daily *A*_cnet_ reached the largest gain of ~12%, 3%, and 2%, respectively (Figure 7(j)).

### 3.6. Erect Leaves, Lower Spike Position, Shorter Plant Height, and Proper Plant Density Benefit Daily *A*_cnet_

**Figure 8:**
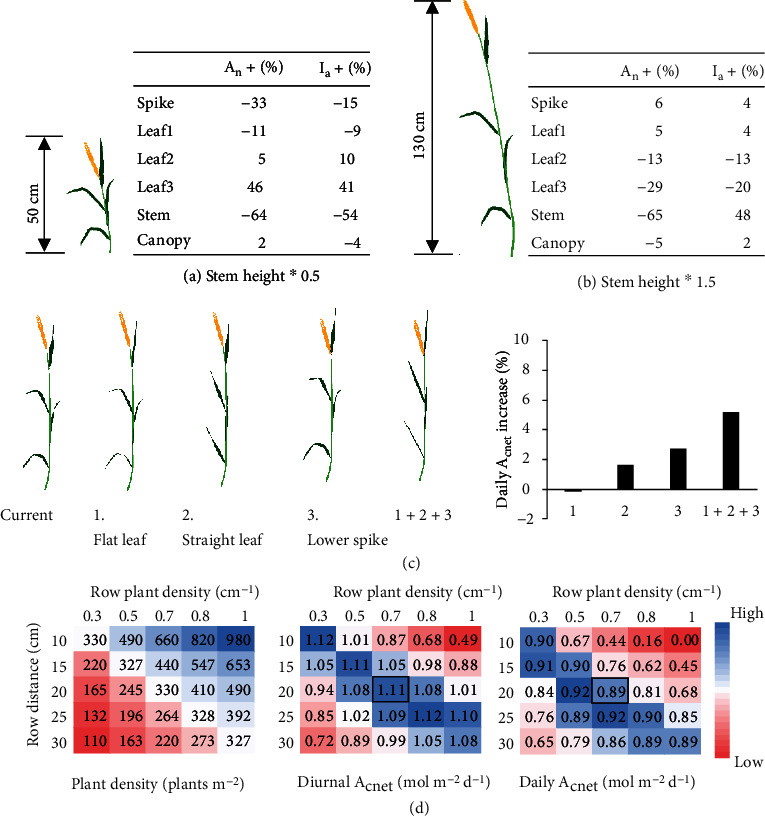
Erect leaves, lower spike, shorter plant height, and proper plant density benefit canopy photosynthesis at the heading stage. (a–b) Responses of daily net tissue and canopy photosynthesis and light absorption to a 50% decreased stem height (a) or a 50% increased stem height (b). (c) The responses of daily net canopy photosynthesis to different manipulations of plant architecture: (1) leaving out the twisting feature of leaves; (2) straightening leaves along their initial direction at leaf base; (3) coinciding the base points of the spike and the uppermost leaf; and (4) a combination of (1), (2), and (3). (d) Plant density, diurnal *A*_cnet_, and daily *A*_cnet_ under different combinations of row distance and plant density in a row.

During conventional high yielding crop breeding in cereals, a major focus is improving canopy architecture at heading. To quantify the impact of canopy architecture on canopy photosynthesis, we evaluated effects of changing major plant architectural parameters on daily *A*_cnet_ at the heading stage. We first evaluated the effect of modifying plant height on daily *A*_cnet_. Plant height is modified to be either 0.5-fold or 1.5-fold of the default height at the heading stage (Figures 8(a) and 8(b)). In both scenarios, the size and shape of leaves and spikes and the relative position of leaves on the stem were not changed. Simulations show that stem height influences light absorption and daily net photosynthesis of all tissues. For example, when stem height was reduced to 0.5-fold of its original value, daily net photosynthesis of the spike, the flag leaf and the stem were reduced by 33%, 11%, and 64%, respectively, due to less light absorption by these tissues, whereas daily net photosynthesis and light absorption of the lower leaves were increased (Figure 8(a)).

The different change trends of light absorption of different tissues can be understood by taking into account that when stem height was reduced, the relative height of the upper tissues became lower, while the relative height of the lower tissues became higher in the canopy. In terms of stem, its reduction in light absorption was a result of its reduced length and surface area. In contrast, when the plant height was increased to be 1.5 fold of its default value, the net photosynthesis of spike and the flag leaf were increased, while the net photosynthesis of lower leaves and stem were decreased (Figure 8(b)). For stem, although it had increased light absorption (+48%), it decreased in net photosynthesis (-65%), which was a result of a higher increase in respiration than in photosynthesis with longer stem length.

In brief, at the canopy level, although a decrease of the stem height by 50% decreased total canopy light absorption by 4%, it increased daily *A*_cnet_ by 2% as a result of increased photosynthetic CO_2_ uptake of lower leaves, whereas although an increase of stem height by 50% increased canopy light absorption by 2%, it decreased daily *A*_cnet_ by 5% as a result of a dramatic decrease in photosynthesis of plant tissues below the flag leaf (Figures 8(a) and 8(b)). On the other hand, the predicted small difference in canopy photosynthesis between dwarf (~50 cm in plant height), semidwarf (~90 cm in plant height), and tall (~130 cm in plant height) wheat cultivars at the heading stage was consistent with experimental data after spike emergence in winter wheat [43].

In rice, the modern high yielding cultivars usually have erect leaves and a short distance between the base of the flag leaf and the base of the spike. We tested whether incorporation of these morphological features can also benefit canopy photosynthesis in wheat. Specifically, we reconstructed new plant types with the following manipulations: (1) flattening leaves, i.e., leaving out the twisting feature of leaves; (2) straightening leaves along their initial direction at leaf base; (3) overlapping the base points of the spike and the uppermost leaf; and (4) a combination of (1), (2) and (3). The 1^st^ modification hardly influenced, while the 2^nd^ and the 3^rd^ modification increased daily *A*_cnet_ by 1.6% and 2.7%, respectively. Remarkably, a combination of the (1), (2) and (3) modifications increased the daily *A*_cnet_ by 5.2% (Figure 8(c)). This result indicates that a lower spike position and straight and erect leaves could benefit wheat daily *A*_cnet_.

The tiller number per unit ground area is another major factor influencing crop canopy architecture, photosynthesis, crop biomass, and yield. Here we evaluated diurnal and daily *A*_cnet_ at the heading stage with different combinations of row distance (10, 15, 20, 25, 30 cm) and distance between plants in a row (0.33, 0.49, 0.66, 0.82, and 0.98 cm). All plant architectural features were kept constant.

First, diurnal/daily *A*_cnet_ is low under both high and low tiller numbers per unit ground area (Figure 8(d)). Specifically, the highest diurnal net photosynthesis was observed with a planting pattern of 10 cm∗0.33 cm^−1^ and 25 cm∗0.82 cm^−1^ (the photosynthesis is 0.9% higher than the current planting pattern of 20 cm∗0.66 cm^−1^), while the highest daily *A*_cnet_ was predicted for a canopy with a planting pattern of 20 cm∗0.49 cm^−1^ (the photosynthesis is 3.2% higher than the current planting pattern). However, a planting pattern of 20 cm∗0.49 cm^−1^ means 25% less spikes per m^2^ land, i.e., lower grain sink capacity. Among the planting patterns which have equal or higher grain sink capacity, daily *A*_cnet_ of the current planting pattern is near optimal, which is 99% of the highest daily *A*_cnet_ (the highest daily *A*_cnet_ are achieved with planting patterns of 10 cm∗0.33 cm^−1^ and 25 cm∗0.82 cm^−1^).

These results indicate that, on one hand, a proper plant density is needed to balance tissue photosynthetic gain and respiratory cost; on the other hand, the optimal planting patterns may be diverse; i.e., there are different combinations of row distance and plant density in a row which can achieve similar high canopy photosynthesis and grain sink capacity.

## 4. Discussion

### 4.1. Development of a Complete Model for Canopy Photosynthesis

Although a number of canopy photosynthesis models have been developed to date, they usually lack a detailed description of canopy architecture, especially for nonfoliar tissues [9, 44, 45]. However, as we have shown above, spike and stem are also photosynthetically active during grain filling and are important contributors of canopy photosynthesis. Therefore, these models cannot be used to study canopy photosynthesis at the grain filling stage [39].

Here we present a complete model, the 3dCAP-wheat, which explicitly simulates the photosynthesis of both foliar tissues and nonfoliar tissues (i.e., stem and spike). As a result, the 3dCAP-wheat can be used to enable quantitative study of canopy photosynthesis across the whole growing season (Figure 4). Moreover, this model can be used to estimate the contribution of different features to total canopy photosynthesis, identify options to gain higher photosynthetic efficiency, and define optimal combinations for further enhancement of canopy photosynthesis. In addition, to enable efficient model parameterization, we have developed a pipeline to measure architectural and physiological parameters as model input. To ease the application and further development of the model by the community, we have made the source code, together with the user manual, freely available for noncommercial use.

There are several potential limitations of this approach. Firstly, the 3dCAP-wheat model needs detailed measurement of plant architecture and photosynthetic physiology of individual plant tissues, which can be more time-consuming than leaf area index-based methods. Secondly, in current model, we do not consider photosynthesis under stresses, e.g., drought, salt, extreme temperature, and biotic stresses. Thirdly, in current model, we do not simulate the dynamic plant growth and the influence of source sink relationship on photosynthesis, which has been done in another independent work (Chang et al., unpublished data).

### 4.2. Morphological Features of Wheat Canopy for Maximizing Photosynthetic Efficiency

Enhancing crop yield potential is a major focus of modern crop breeding. However, defining the optimal combination of traits still represents a major challenge for breeders [46, 47]. The model and related method for model parameterization and analysis reported here can be used as a generic approach to identify options to improve canopy photosynthesis. In this pilot study, an elite Chinese wheat cultivar, N22, is used as an example to show how to use the 3dCAP-wheat for identifying options for further improvement. We have identified many features which are largely consistent with current breeding experience, suggesting that this model can be used to support further wheat crop improvement. Here we discuss these features and their relevance to breeding and discuss them with the current understanding of their role in canopy photosynthesis:

Morphologically, we found that the daily *A*_cnet_ at the heading stage can increase 5% by having more erect leaves and lowering the spike basal position to the base of the flag leaf, if other morphological and physiological traits remain unchanged (Figure 8(c)). In contrast, neither increase in spike length, grain size, awn length, spike number (beyond a certain optimal value) nor increase in plant height can further improve canopy photosynthesis at the heading stage (Figures 6(a)–6(d) and 8(b)).

Taking a tall plant (e.g. 130 cm) as an example, we have shown that regardless of its higher risk of lodging and its more biomass partitioned into stem (which is less photosynthetically active), more light in the canopy would be intercepted by spike and stem due to elongated internode length below the spike (so higher relative spike position in the canopy) and increased stem surface area (Figure 8(b)). This results in a slight reduction (5%) in canopy photosynthesis. Given that taller plants tend to have even bigger spikes, this reduction in photosynthetic source and increase in carbohydrate sink would result in an imbalanced plant source sink relationship.

In line with this analysis, for wheat breeding in the last century, plant height keeps decreasing both in China [48] and in Italy [49]; and it was found that canopy photosynthesis at the heading stage did not significantly differ between dwarf, semidwarf, and tall cultivars [43]. Together with our simulation results, we conclude that tall cultivars do not have advantage in canopy photosynthesis during the grain filling period.

Similarly, during a century's high-yield wheat breeding, neither spike length [49], awn length [30], nor spike number changed significantly [48], whereas the grain size (kernel weight) significantly increased [48, 49]. It is obvious that, to increase grain yield potential, breeders have to increase one or multiple grain yield-related components, i.e., spike number, spike length, and grain size (and/or filled spikelet number). The question is why breeders preferred to increase grain size (and/or filled spikelet number) rather than the other two? Intriguingly, from theoretical calculation of canopy photosynthesis, we found that increasing the three traits may all decrease daily *A*_cnet_, but the magnitude of this decrease differs. Specifically, if we increase spike number 33% (from 328 to 435 m^−2^), the *A*_cnet_ will decrease 27%; if we increase spike length 33% (from 9 to 12 cm), the daily *A*_cnet_ will decrease 7%; if we increase spikelet size 32% (from 31 to 41 mg), the daily *A*_cnet_ will decrease only 4% (Figure 6(a)–6(c)). Accordingly, increasing grain size (and/or filled spikelet number) may be the best choice of breeders to maintain the canopy photosynthetic capacity while increasing the grain yield potential.

### 4.3. Physiological Features of Wheat Canopy for Maximizing Photosynthetic Efficiency

Physiologically, improvement of photosynthetic activity is an important approach to further enhancement of crop yield [32]. A key issue is how much canopy photosynthesis can be increased from the increased tissue-level photosynthetic capacity [9]. Here we found that for the wheat cultivar N22 at the heading stage, increasing leaf *A*_max_net_, or *Φ*_CO2_, or both by 10% can lead to an increase of daily net canopy photosynthesis of 4.9%, or 6.4%, or 12%, respectively (Figure 7(j)).

However, simultaneous increase of leaf photosynthetic and respiratory rates by increasing tissue nitrogen contents will convert into much less (or even negative) net canopy photosynthesis enhancement (Figures 7(a)–7(f)). These nonlinear responses of daily *A*_cnet_ to leaf nitrogen content may partially underlie the frequently observed quadratic relationship between the nitrogen application rates and the plant biomass and yield [50–52]. Given that the *A*_max_leaf_ values are in a range of 20-35 *μ*mol m^−2^ s^−1^ for most of the wheat cultivars grown under a wide range of conditions, our simulation predicted weak or no correlation between *A*_max_leaf_ and daily *A*_cnet_ in this range (Figures 7(g)–7(i)). Again, this is related to the expected higher respiratory cost in canopies with higher *A*_max_leaf_. These analyses may at least partially explain why there is no significant correlation between *A*_max_leaf_ and plant biomass [53]. These results also highlight the importance of enhancing photosynthesis without the associated increase in respiration.

Spike photosynthesis has long been regarded as an important player in cereal crop yield formation, especially in wheat and barley [11, 13, 54–56]. When spike photosynthesis is estimated with different methodologies, in different wheat lines and under different growth conditions, it was found that spike photosynthesis contributed 4.4% to 97% of the grain yield [39]. However, these methods either did not consider the photosynthetic activity on a surface area basis [14] or did not consider the light interception of the spike [17] or did not consider the CO_2_ source of spike photosynthesis. Therefore, these methods cannot quantitatively assess the photosynthetic roles of leaves and spikes in a canopy.

Here, by comprehensive measurement and modeling the structure and photosynthetic physiology of both the spikes and other organs in a canopy, we are able to show that at heading, the spike has a light interception of ~28%, which is comparable to that of a flag leaf, and a LUE of ~2.5%, which is much lower than that of a flag leaf. More importantly, ~50% of the CO_2_ for spike photosynthesis is from spike respiration. As a result, in a closed canopy, removal of spikes would “transfer” the light intercepted by the spikes to the leaves, which have much higher photosynthetic activity than the spikes (on a surface area basis). And finally, the canopy (net) photosynthesis would be increased rather than decreased. And for the same reason, we suggest that in the direction of increasing spike photosynthesis, researchers should focus on increasing the photosynthetic activity of glumes and awns rather than focus on increase spike number, spike size, and/or awn size (Figure 6).

Most efforts so far on improving photosynthesis have focused on foliar tissues, and many effective options to improve leaf photosynthesis have been identified [5, 8, 57–60]. The effectiveness of these approaches in increasing spike photosynthesis needs to be tested. Furthermore, since genetic variation of spike photosynthesis may be independent of flag leaf photosynthesis [17], it is also timely to mine and exploit alleles controlling spike photosynthesis as well.

In this regard, our custom-built P-Chamber [16] could be served as a feasible phenotyping tool for large-scale spike photosynthetic activity screening. Specifically, for each spike, the measurement of *A*_1500_ and *R*_*d*_ takes in total<10 min in a temperature-controlled greenhouse. Using four P-Chambers, one could measure a large breeding panel of 150 genotypes∗3 repetitions in 3 days (6 hours a day). The area of the spikes can be measured by sampling and imaging the spikes (see in the Materials and methods).

Lastly, while flag leaf photosynthesis has been described as a key trait to improve cereal yields, recent research has suggested the importance of middle and bottom layers of a canopy to radiation use efficiency, nonphotochemical quenching, and crop yield in wheat [61], rice [62], and cotton [63]. Here, we emphasize that other leaves besides the flag leaf are indeed key players of canopy photosynthesis, which contribute ~40%/~35% of net/gross canopy photosynthesis at the heading stage and~40%/~20% of net/gross canopy photosynthesis at the milking stage. Moreover, these lower leaves, most of which are under light limited conditions during the day, have the highest LUE in the canopy, compared with the flag leaf, spike, and stem (Figures 5(d) and 5(f); Supplementary Fig. 5d, f).

In summary, the analysis from this modeling study and also earlier experimental results together suggest that features for a wheat ideotype for enhanced photosynthetic efficiency during the grain filling season may include (1) more erect leaves, (2) lower spike position in the canopy, (3) semidwarf plant height, (4) proper plant density, (5) increased photosynthetic activity of both foliar and nonfoliar tissues, and (6) lower leaf respiratory activity.

## Data Availability

Source code used for this study, together with the user manual, are freely available for noncommercial use at https://github.com/rootchang/3dCAP-wheat.

## Supplementary Materials

Supplementary Materials Supplementary Figure 1: characterizing nonfoliar photosynthesis and canopy photosynthesis using custom-built instruments. Supplementary Figure 2: an illustration of parameterization and validation of the nonfoliar photosynthesis model. Supplementary Figure 3: using a transparent cuvette to monitor 24-hour gas exchange rate of a spike. Supplementary Figure 4: light distribution within the canopy on a sunny day at 12 o'clock at the heading stage. Supplementary Figure 5: a landscape of photosynthetic characteristics of different foliar and nonfoliar tissues in the canopy. Supplementary Figure 6: weather information on typical sunny days of the tillering (a), heading (b), and grain milk (c) stages. Supplementary Figure 7: relative change of tissue daily net photosynthesis (*A*_*n*_) and light absorption (*I*_*a*_) with different awn length.

## References

[1] Zelitch I. (1982). The close relationship between net photosynthesis and crop yield. *Bioscience*.

[2] Bailey-Serres J., Parker J. E., Ainsworth E. A., Oldroyd G. E. D., Schroeder J. I. (2019). Genetic strategies for improving crop yields. *Nature*.

[3] Simkin A. J., López-Calcagno P. E., Raines C. A. (2019). Feeding the world: improving photosynthetic efficiency for sustainable crop production. *Journal of Experimental Botany*.

[4] Zhu X.-G., Long S. P., Ort D. R. (2010). Improving photosynthetic efficiency for greater yield. *Annual Review of Plant Biology*.

[5] Kromdijk J., Głowacka K., Leonelli L. (2016). Improving photosynthesis and crop productivity by accelerating recovery from photoprotection. *Science*.

[6] Perveen S., Qu M., Chen F. (2020). Overexpression of maize transcription factor mEmBP-1 increases photosynthesis, biomass, and yield in rice. *Journal of Experimental Botany*.

[7] Qu M., Zheng G., Essmine J. (2017). Leaf photosynthetic parameters related to biomass accumulation in a global rice diversity survey. *Plant Physiology*.

[8] South P. F., Cavanagh A. P., Liu H. W., Ort D. R. (2019). Synthetic glycolate metabolism pathways stimulate crop growth and productivity in the field. *Science*.

[9] Wu A., Hammer G. L., Doherty A., von Caemmerer S., Farquhar G. D. (2019). Quantifying impacts of enhancing photosynthesis on crop yield. *Nature Plants*.

[10] Hu L., Zhang Y., Xia H. (2019). Photosynthetic characteristics of non-foliar organs in main C_3_ cereals. *Physiologia Plantarum*.

[11] Parry M. A. J., Reynolds M., Salvucci M. E. (2011). Raising yield potential of wheat. II. Increasing photosynthetic capacity and efficiency. *Journal of Experimental Botany*.

[12] Sanchez-Bragado R., Molero G., Reynolds M. P., Araus J. L. (2016). Photosynthetic contribution of the ear to grain filling in wheat: a comparison of different methodologies for evaluation. *Journal of Experimental Botany*.

[13] Sanchez-Bragado R., Vicente R., Molero G., Serret M. D., Maydup M. L., Araus J. L. (2020). New avenues for increasing yield and stability in C_3_ cereals: exploring ear photosynthesis. *Current Opinion in Plant Biology*.

[14] Sanchez-Bragado R., Molero G., Reynolds M. P., Araus J. L. (2014). Relative contribution of shoot and ear photosynthesis to grain filling in wheat under good agronomical conditions assessed by differential organ *δ*13C. *Journal of Experimental Botany*.

[15] Xu M., Shi N., Li Q., Mi H. (2014). An active supercomplex of NADPH dehydrogenase mediated cyclic electron flow around photosystem I from the panicle chloroplast of *Oryza sativa*. *Acta Biochimica et Biophysica Sinica*.

[16] Chang T.-G., Song Q.-F., Zhao H.-L. (2020). An in situ approach to characterizing photosynthetic gas exchange of rice panicle. *Plant Methods*.

[17] Molero G., Reynolds M. P. (2020). Spike photosynthesis measured at high throughput indicates genetic variation independent of flag leaf photosynthesis. *Field Crops Research*.

[18] Long S. P., Zhu X. G., Naidu S. L., Ort D. R. (2006). Can improvement in photosynthesis increase crop yields?. *Plant, Cell and Environment*.

[19] Sarlikioti V., de Visser P. H. B., Buck-Sorlin G. H., Marcelis L. F. M. (2011). How plant architecture affects light absorption and photosynthesis in tomato: towards an ideotype for plant architecture using a functional–structural plant model. *Annals of Botany*.

[20] Song Q. F., Zhang G. L., Zhu X. G. (2013). Optimal crop canopy architecture to maximise canopy photosynthetic CO_2_ uptake under elevated CO_2_-a theoretical study using a mechanistic model of canopy photosynthesis. *Functional Plant Biology*.

[21] Donald C. M. (1968). The breeding of crop ideotypes. *Euphytica*.

[22] Maddonni G. A., Otegui M. E., Cirilo A. G. (2001). Plant population density, row spacing and hybrid effects on maize canopy architecture and light attenuation. *Field Crops Research*.

[23] Setter T., Conocono E., Egdane J., Kropff M. (1995). Possibility of increasing yield potential of rice by reducing panicle height in the canopy. I. Effects of panicles on light interception and canopy photosynthesis. *Functional Plant Biology*.

[24] Yuan L. (2017). Progress in super-hybrid rice breeding. *The Crop Journal*.

[25] Peng S., Khush G. S., Virk P., Tang Q., Zou Y. (2008). Progress in ideotype breeding to increase rice yield potential. *Field Crops Research*.

[26] Richards R. A., Cavanagh C. R., Riffkin P. (2019). Selection for erect canopy architecture can increase yield and biomass of spring wheat. *Field Crops Research*.

[27] Sakamoto T., Morinaka Y., Ohnishi T. (2006). Erect leaves caused by brassinosteroid deficiency increase biomass production and grain yield in rice. *Nature Biotechnology*.

[28] Tian J. G., Wang C. L., Xia J. L. (2019). Teosinte ligule allele narrows plant architecture and enhances high-density maize yields. *Science*.

[29] Masoudi B., Mardi M., Hervan E. M. (2019). Study of QTLs linked to awn length and their relationships with chloroplasts under control and saline environments in bread wheat. *Genes & Genomics*.

[30] Maydup M. L., Antonietta M., Graciano C., Guiamet J. J., Tambussi E. A. (2014). The contribution of the awns of bread wheat (Triticum aestivum L.) to grain filling: Responses to water deficit and the effects of awns on ear temperature and hydraulic conductance. *Field Crops Research*.

[31] Chang T.-G., Chang S., Song Q.-F., Perveen S., Zhu X.-G. (2019). Systems models, phenomics and genomics: three pillars for developing high-yielding photosynthetically efficient crops. *in silico Plants*.

[32] Long S. P., Marshall-Colon A., Zhu X.-G. (2015). Meeting the global food demand of the future by engineering crop photosynthesis and yield potential. *Cell*.

[33] Marshall-Colon A., Long S. P., Allen D. K. (2017). Crops in silico: generating virtual crops using an integrative and multi-scale modeling platform. *Frontiers in Plant Science*.

[34] van Ittersum M. K., Leffelaar P. A., van Keulen H., Kropff M. J., Bastiaans L., Goudriaan J. (2003). On approaches and applications of the Wageningen crop models. *European Journal of Agronomy*.

[35] de Wit C. T. (1965). *Photosynthesis of Leaf Canopies*.

[36] De Pury D., Farquhar G. (1997). Simple scaling of photosynthesis from leaves to canopies without the errors of big-leaf models. *Plant, Cell and Environment*.

[37] Townsend A. J., Retkute R., Chinnathambi K. (2018). Suboptimal acclimation of photosynthesis to light in wheat canopies. *Plant Physiology*.

[38] Truong S. K., McCormick R. F., Rooney W. L., Mullet J. E. (2015). Harnessing genetic variation in leaf angle to increase productivity of *Sorghum bicolor*. *Genetics*.

[39] Zhang M., Gao Y., Zhang Y. (2020). The contribution of spike photosynthesis to wheat yield needs to be considered in process-based crop models. *Field Crops Research*.

[40] Feret J. B., François C., Asner G. P. (2008). PROSPECT-4 and 5: advances in the leaf optical properties model separating photosynthetic pigments. *Remote Sensing of Environment*.

[41] Uddling J., Gelang-Alfredsson J., Piikki K., Pleijel H. (2007). Evaluating the relationship between leaf chlorophyll concentration and SPAD-502 chlorophyll meter readings. *Photosynthesis Research*.

[42] Song Q., Xiao H., Xiao X., Zhu X.-G. (2016). A new canopy photosynthesis and transpiration measurement system (CAPTS) for canopy gas exchange research. *Agricultural and Forest Meteorology*.

[43] Gent M. P. N. (1995). Canopy light interception, gas exchange, and biomass in reduced height isolines of winter wheat. *Crop Science*.

[44] Ikawa H., Chen C. P., Sikma M. (2018). Increasing canopy photosynthesis in rice can be achieved without a large increase in water use—a model based on free-air CO2 enrichment. *Global Change Biology*.

[45] Wang Y., Song Q. F., Jaiswal D., de Souza A. P., Long S. P., Zhu X. G. (2017). Development of a three-dimensional ray-tracing model of sugarcane canopy photosynthesis and its application in assessing impacts of varied row spacing. *Bioenergy Research*.

[46] Chang T.-G., Zhao H., Wang N. (2019). A three-dimensional canopy photosynthesis model in rice with a complete description of the canopy architecture, leaf physiology, and mechanical properties. *Journal of Experimental Botany*.

[47] Yuan W., Peng S., Cao C. (2011). Agronomic performance of rice breeding lines selected based on plant traits or grain yield. *Field Crops Research*.

[48] Qin X., Zhang F., Liu C. (2015). Wheat yield improvements in China: past trends and future directions. *Field Crops Research*.

[49] De Vita P., Nicosia O. L. D., Nigro F. (2007). Breeding progress in morpho-physiological, agronomical and qualitative traits of durum wheat cultivars released in Italy during the 20th century. *European Journal of Agronomy*.

[50] Koenig R. T., Cogger C. G., Bary A. I. (2011). Dryland winter wheat yield, grain protein, and soil nitrogen responses to fertilizer and biosolids applications. *Applied and Environmental Soil Science*.

[51] Shapiro C. A., Wortmann C. S. (2006). Corn response to nitrogen rate, row spacing, and plant density in eastern Nebraska. *Agronomy Journal*.

[52] Yang J. (2015). Approaches to achieve high grain yield and high resource use efficiency in rice. *Frontiers of Agricultural Science and Engineering*.

[53] Wells R., Meredith W. R., Williford J. R. (1986). Canopy photosynthesis and its relationship to plant productivity in near-isogenic cotton lines differing in leaf morphology. *Plant Physiology*.

[54] Faralli M., Lawson T. (2020). Natural genetic variation in photosynthesis: an untapped resource to increase crop yield potential?. *The Plant Journal*.

[55] Furbank R. T., Sharwood R., Estavillo G. M., Silva-Perez V., Condon A. G. (2020). Photons to food: genetic improvement of cereal crop photosynthesis. *Journal of Experimental Botany*.

[56] Tambussi E. A., Maydup M. L., Carrión C. A., Guiamet J. J., Araus J. L. (2021). Ear photosynthesis in C_3_ cereals and its contribution to grain yield: methodologies, controversies, and perspectives. *Journal of Experimental Botany*.

[57] Ambavaram M. M. R., Basu S., Krishnan A. (2014). Coordinated regulation of photosynthesis in rice increases yield and tolerance to environmental stress. *Nature Communications*.

[58] Simkin A. J., Mcausland L., Headland L. R., Lawson T., Raines C. A. (2015). Multigene manipulation of photosynthetic carbon assimilation increases CO2 fixation and biomass yield in tobacco. *Journal of Experimental Botany*.

[59] Simkin A. J., Mcausland L., Lawson T., Raines C. A. (2017). Over-expression of the RieskeFeS protein increases electron transport rates and biomass yield. *Plant Physiology*.

[60] Wang Y., Noguchi K., Ono N., Inoue S., Terashima I., Kinoshita T. (2014). Overexpression of plasma membrane H^+^-ATPase in guard cells promotes light-induced stomatal opening and enhances plant growth. *Proceedings of the National Academy of Sciences of the United States of America*.

[61] Robles-Zazueta C. A., Pinto F., Molero G., Foulkes M. J., Reynolds M. P., Murchie E. H. (2022). Prediction of photosynthetic, biophysical, and biochemical traits in wheat canopies to reduce the phenotyping bottleneck. *Frontiers in Plant Science*.

[62] Foo C. C., Burgess A. J., Retkute R., Tree-Intong P., Ruban A. V., Murchie E. H. (2020). Photoprotective energy dissipation is greater in the lower, not the upper, regions of a rice canopy: a 3D analysis. *Journal of Experimental Botany*.

[63] Yao H. S., Zhang Y. L., Yi X. P., Zhang X. J., Zhang W. F. (2016). Cotton responds to different plant population densities by adjusting specific leaf area to optimize canopy photosynthetic use efficiency of light and nitrogen. *Field Crops Research*.

