# Supplementary material for: 3dCAP-Wheat: An Open-Source Comprehensive Computational Framework Precisely Quantifies Wheat Foliar, Nonfoliar, and Canopy Photosynthesis
Source: Plant Phenomics. 2022 Jul 21;2022:9758148. doi: 10.34133/2022/9758148 (PMC9394111; doi:10.34133/2022/9758148)
Supplement: Supplementary Materials — Supplementary Figure 1: characterizing nonfoliar photosynthesis and canopy photosynthesis using custom-built instruments. Supplementary Figure 2: an illustration of parameterization and validation of the nonfoliar photosynthesis model. Supplementary Figure 3: using a transparent cuvette to monitor 24-hour gas exchange rate of a spike. Supplementary Figure 4: light distribution within the canopy on a sunny day at 12 o'clock at the heading stage. Supplementary Figure 5: a landscape of photosynthetic characteristics of different foliar and nonfoliar tissues in the canopy. Supplementary Figure 6: weather information on typical sunny days of the tillering (a), heading (b), and grain milk (c) stages. Supplementary Figure 7: relative change of tissue daily net photosynthesis (An) and light absorption (Ia) with different awn length. [file 9758148.f1.docx]

# Supplementary information for

**3dCAP-wheat: an open-source comprehensive computational framework precisely quantifies wheat foliar, non-foliar and canopy photosynthesis**

Tian-Gen Chang^1#^, Zai Shi^1#^, Honglong Zhao^1^, Qingfeng Song^1^, Zhonghu He^2,4^, Jeroen Van Rie^3^, Bart Den Boer^3^, Alexander Galle^3^, Xin-Guang Zhu^1,*^

^1^National Key Laboratory for Plant Molecular Genetics, Center for Excellence in Molecular Plant Sciences, Chinese Academy of Sciences, Shanghai 200032, China

^2^Insitute of Crop Sciences, Chinese Academy of Agricultural Sciences, Beijing 100081, China

^3^BASF Belgium Coordination Center – Innovation Center Gent, Technologiepark-Zwijnaarde 101, 9052 Gent, Belgium

^4^International Maize and Wheat Improvement Center (CIMMYT) China Office, c/o Chinese Academy of Agricultural Sciences, Beijing 100081, China

^#^These authors contributed equally to this work

^*^Corresponding author : [zhuxg@cemps.ac.cn](mailto:zhuxg@cemps.ac.cn)

Telephone number for corresponding author: +86-21-54924163


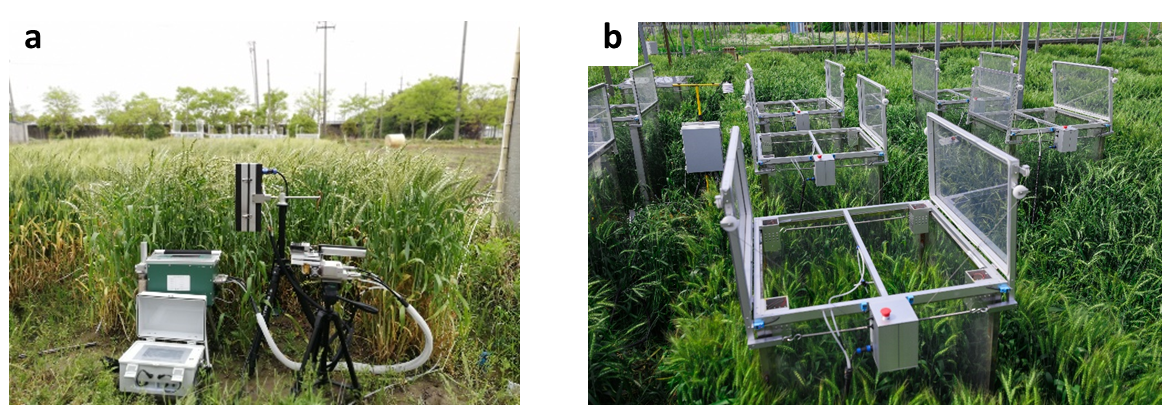


**Supplementary Figure 1.** **Characterizing non-foliar photosynthesis and canopy photosynthesis using custom-built instruments.** **a**, measuring wheat spike photosynthetic light response (*A*-Q) curve using the P-Chamber. **b**, monitoring the daily dynamic canopy gas exchange rate using the canopy photosynthesis and transpiration system.


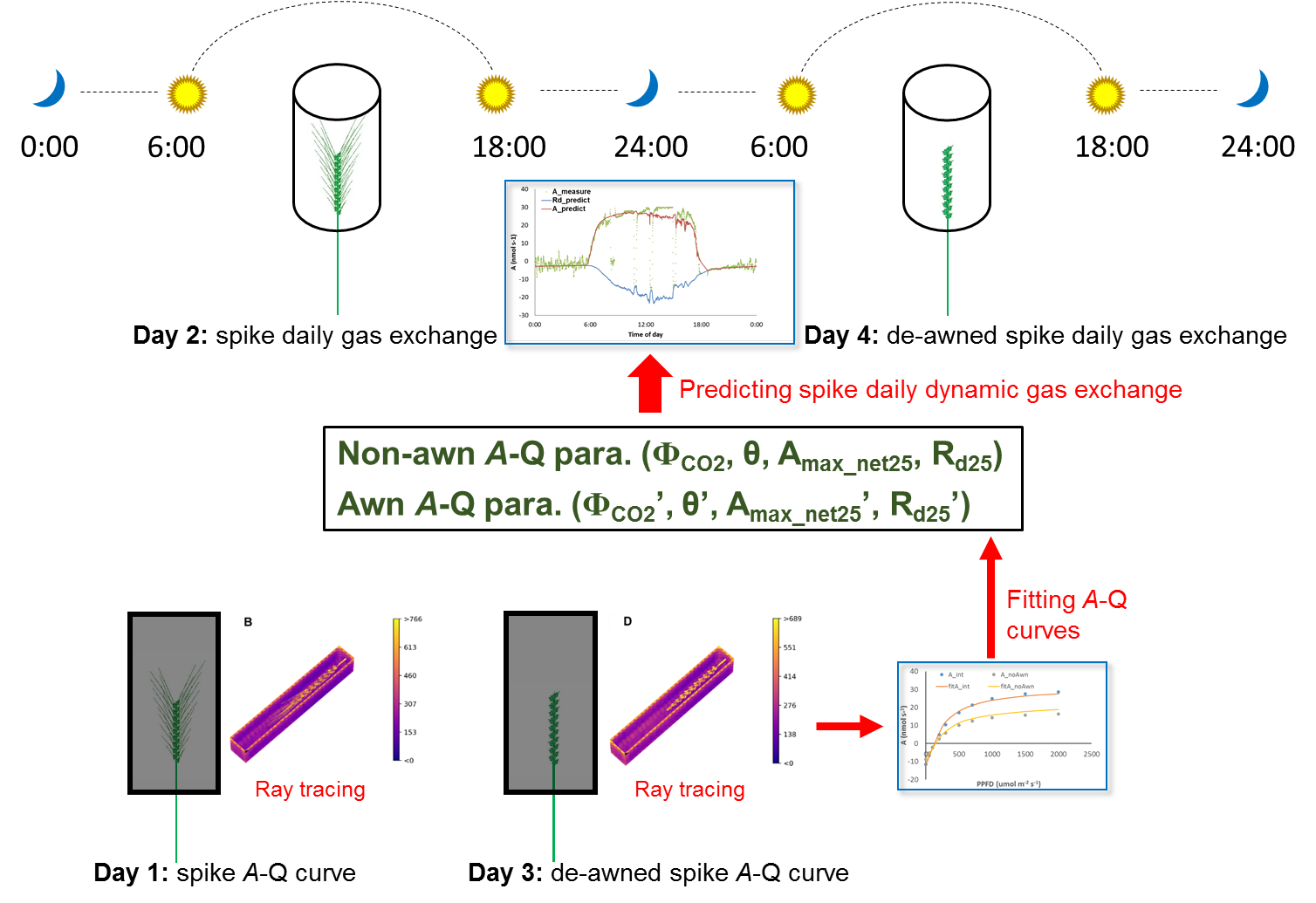


**Supplementary Figure 2.** **An illustration of parameterization and validation of the non-foliar photosynthesis model.** See detailed description in **Materials and methods**.


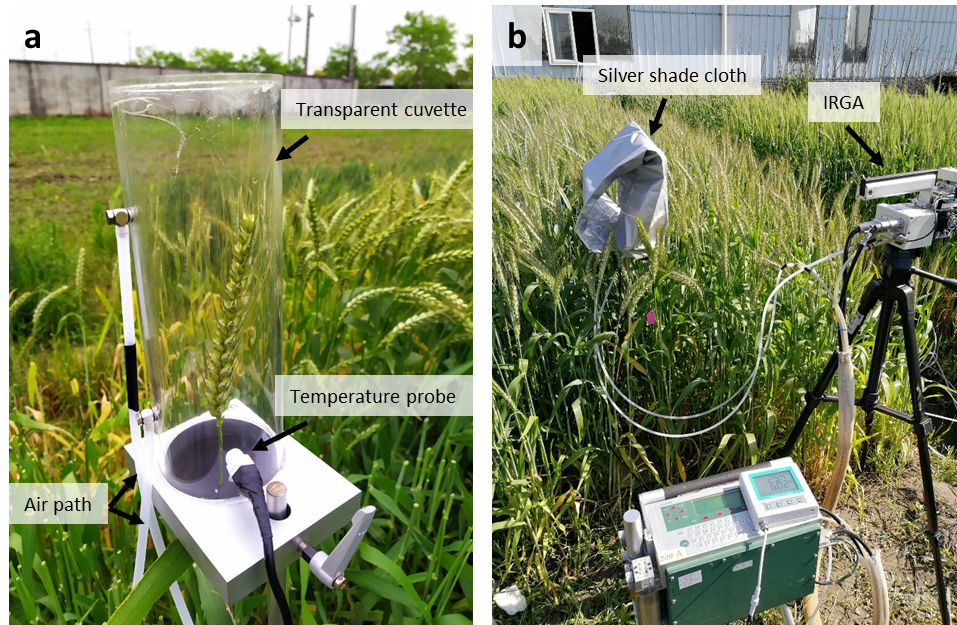


**Supplementary Figure 3.** **Using a transparent cuvette to monitor 24-hour gas exchange rate of a spike.** A transparent cuvette equipped with a temperature probe (**a**) for measuring the 24-hour gas exchange rate of a spike by connecting to an infrared gas analyzer (IRGA, **b**). At certain time points in the daytime, a silver shade cloth is used to cover the spike transiently to measure the respiratory rate (b).


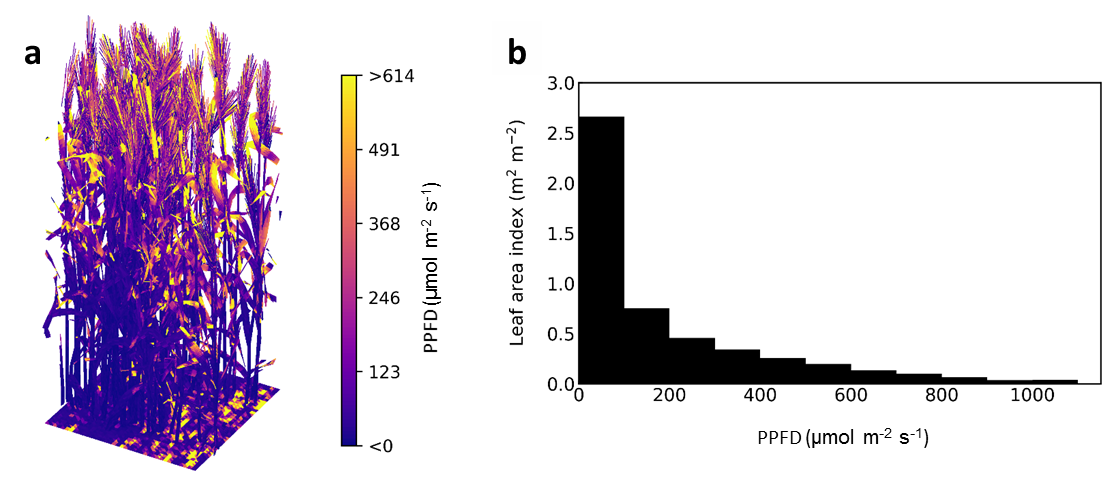


**Supplementary Figure 4.** **Light distribution within the canopy on a sunny day at 12 o’clock at the heading stage.** **a**, an illustration of light levels in the canopy. **b**, distribution of the amount of leaf area receiving different light intensities.


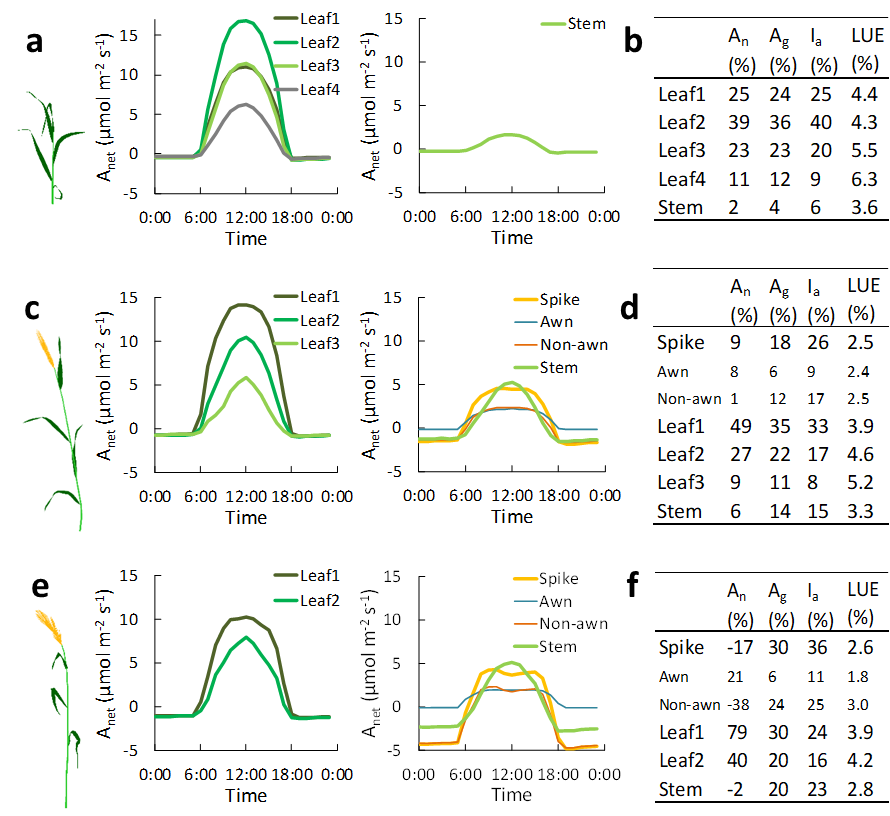


**Supplementary Figure 5. A landscape of photosynthetic characteristics of different foliar and non-foliar tissues in the canopy.** The wheat cultivar Y20 was used. The 24-hour dynamic net gas exchange rate of different tissues in the canopy at the tillering (**a**), heading (**c**) and grain milk (**e**) stages on typical sunny days. The daily net photosynthesis, daily gross photosynthesis, daily light absorption and daily light use efficiency (LUE) of different tissues at the tillering (**b**), heading (**d**) and grain milk (**f**) stages on typical sunny days. A_n_ (%), the ratio between tissue daily net photosynthesis and canopy net gross photosynthesis; A_g_ (%), the ratio between tissue daily gross photosynthesis and canopy daily gross photosynthesis; I_a_ (%), the ratio between tissue daily light absorption and canopy daily light absorption; LUE, the ratio between the daily gross photosynthesis and the daily light absorption.


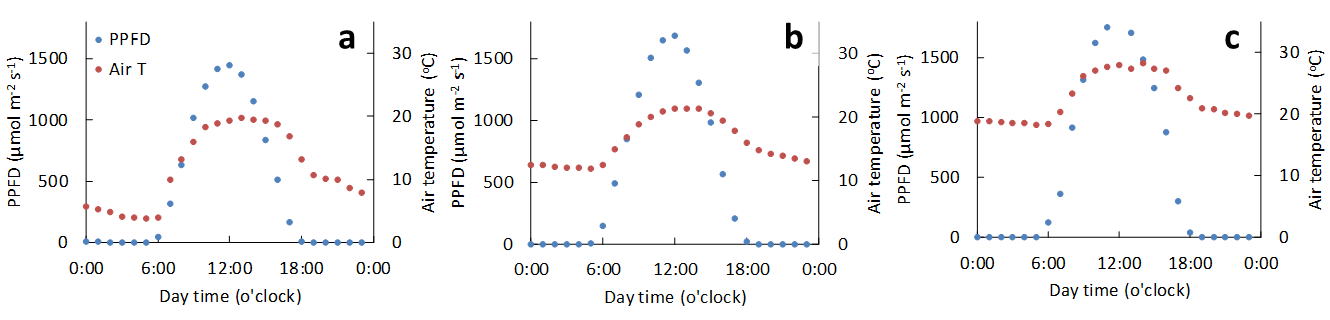


**Supplementary Figure 6. Weather information on typical sunny days of the tillering (a), heading (b) and grain milk (c) stages.** PPFD, photosynthetic photon flux density.


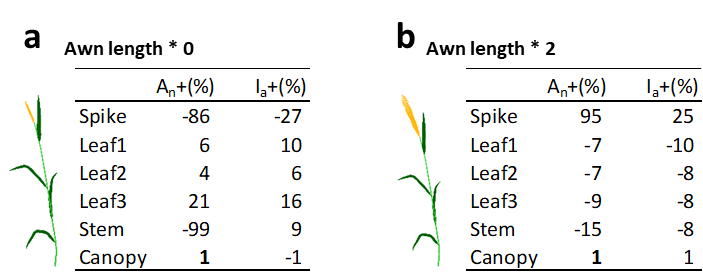


**Supplementary Figure 7. Relative change of tissue daily net photosynthesis (*A*_n_) and light absorption (*I*_a_) with different awn length.** **a**, awn removed (awn length = 0). **b**, awn length doubled.
